# Association between BMI, vitamin D, and estrogen levels in postmenopausal women using adjuvant letrozole: a prospective study

**DOI:** 10.1038/s41523-020-0166-y

**Published:** 2020-06-12

**Authors:** Mitchell J. Elliott, Marguerite Ennis, Kathleen I. Pritchard, Carol Townsley, Dave Warr, Christine Elser, Eitan Amir, Philippe L. Bedard, Lakshmi Rao, Vuk Stambolic, Srikala Sridhar, Pamela J. Goodwin, David W. Cescon

**Affiliations:** 1 Princess Margaret Cancer Centre, Toronto, Canada; 2 University of Toronto, Toronto, Canada; 3 Applied Statistician, Markham, Canada; 4 Sunnybrook Odette Cancer Centre, Toronto, Canada; 5 Women’s College Hospital, Toronto, Canada; 6 Marvelle Koffler Breast Centre, Mount Sinai Hospital, Toronto, Canada; 7 Lunenfeld Tanenbaum Research Institute, Mt. Sinai Hospital, Toronto, Canada

**Keywords:** Breast cancer, Cancer therapy, Medical research

## Abstract

Studies have suggested that women with elevated BMI or 25-OH vitamin D levels may derive less benefit from AIs versus tamoxifen. We prospectively investigated whether high BMI or 25-OH vitamin D levels were associated with higher estrogen levels in post-menopausal women receiving standard adjuvant letrozole (2.5 mg/day). Furthermore, we evaluated whether an increased dose of letrozole resulted in lower serum estrogens in women with BMI > 25 kg/m^2^. Correlation between entry BMI and day 29 serum biomarkers (estrogens, 25-OH vitamin D, insulin, CRP, leptin) was assessed in all patients. On day 29, participants with BMI > 25 kg/m^2^ switched to letrozole 5 mg/day for 4-weeks and blood was drawn upon completion of the study. The change in serum estrogen levels was assessed in these patients (BMI > 25 kg/m^2^). 112 patients completed days 1–28. The Pearson correlations of estradiol and estrone with BMI or serum 25-OH vitamin D levels were near zero (−0.04 to 0.07, *p* = 0.48–0.69). Similar results were obtained for correlation with markers of obesity (insulin, CRP, and leptin) with estradiol and estrone (−0.15 to 0.12; *p* = 0.11–0.82). Thirty-one patients (BMI > 25 kg/m^2^) completed the interventional component; Increasing the dose of letrozole did not further reduce estradiol or estrone levels (change 0.1 and 0.4 pmol/L respectively; *p* = 0.74 and 0.36). There was no observed association between markers of obesity (BMI, insulin, leptin, and CRP), serum 25-OH vitamin D levels and estradiol or estrone levels. Additionally, an increased dose of letrozole did not further reduce estradiol or estrone levels compared to the standard dose.

## Introduction

Aromatase inhibitors (AIs: letrozole, anastrozole, and lexemestane) are the recommended endocrine therapy for most post-menopausal women with estrogen receptor positive (ER+) breast cancer. These agents reduce circulating estradiol and estrone through the inhibition of the enzyme aromatase in adipose tissue, the main source of these hormones in post-menopausal women^1^. The resulting estrogen deprivation reduces ER− dependent signaling in remaining breast cancer cells^2^. However, estrogen suppression is not often complete and may vary between individuals^3^. These differences could be important for the effectiveness of this class of therapy, as it appears that even small amounts of residual estrogens may stimulate breast cancer growth^3,4^. Characterization of host factors that impact the efficacy of estradiol and estrone deprivation may provide opportunities to tailor these interventions.

Women with obesity have higher circulating levels of estrogen due to enhanced peripheral aromatization in fatty tissues. A small study in women initiating AI found that both baseline and on-treatment levels of estrogen were greater at higher levels of BMI, suggesting the possibility of inadequate suppression of aromatase by standard dosing of AI therapy^5^. Retrospective analyses of clinical trials have yielded conflicting outcomes with regards to AI efficacy and obesity; ATAC and ABCSG-12 (which studied anastrozole) demonstrated reduced efficacy of AI therapy compared to tamoxifen in women with obesity, while BIG-1–98 (letrozole) did not^6–8^. In addition to body mass index (BMI), serum markers such as elevated CRP (marker of inflammation)^9^, insulin (marker of insulin resistance)^10^, and leptin (marker of adiposity)^11^, all play a prominent role in obesity physiology and potentially estrogen receptor signaling. Taken together, the impact of elevated BMI and its relationship to AI efficacy remains in question and the potential mechanism underlying any such effect requires further study.

Another possible mediator of AI activity is serum 25-OH vitamin D level. In preclinical studies, 25-OH vitamin D has also been shown to increase aromatase activity in some contexts and upregulate expression of the CYP3A4 drug metabolizing enzyme, which could reduce serum levels of CYP3A4 metabolized drugs (such as letrozole)^12–14^. Furthermore, previously reported retrospective analyses in a cohort of women with early breast cancer identified an association between increased 25-OH vitamin D supplementation and higher residual estradiol or estrone levels in women using AIs^15^. Given the frequent use of vitamin D supplementation and the direct positive correlation between vitamin D consumption and 25-OH vitamin D levels, there is a need for additional data to address the role of this factor on AI activity in the clinical setting.

In this study, we prospectively investigated whether elevated markers of adiposity (BMI, CRP, insulin, and leptin) or serum 25-OH vitamin D (both physiologic and supratherapeutic) levels were associated with higher estradiol and estrone levels in a population of post-menopausal women using adjuvant letrozole. Furthermore, we evaluated whether an increased dose of letrozole resulted in lower estradiol or estrone levels in women with a BMI > 25 kg/m^2^.

## Results

### Patient population

**Fig. 1:**
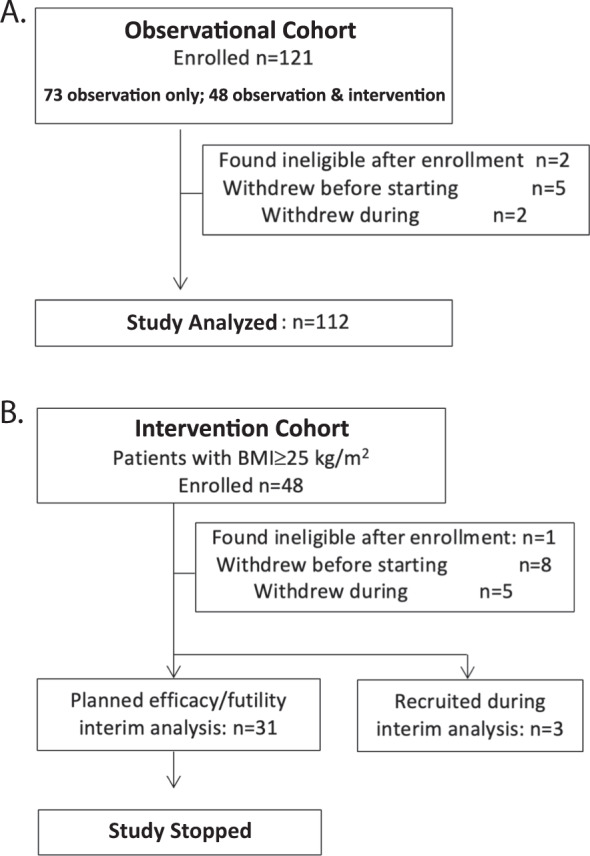
Prospective cohort study design. **a** Prospective open label single arm study to evaluate the association between BMI, serum 25-OH vitamin D and estradiol or estrone levels in women using letrozole as adjuvant endocrine therapy following 1 month of monitored use of letrozole 2.5 mg/day. **b** Interventional single arm study to evaluate the effect of letrozole at 5 mg/day on estradiol and estrone.

One hundred and twenty-one women were recruited from five hospitals in Toronto, Canada between October 2012 and June 2014. Seven patients withdrew before the primary blood collection (Fig. 1a). Two patients were later found to be ineligible and were excluded from analysis; 112 patients completed the initial 28 days of the study.

On day 29, 48 patients with BMI > 25 kg/m^2^ (40% of enrolled patients) continued into the letrozole 5 mg/day intervention (Fig. 1b). Eight eligible patients withdrew before and five withdrew during this phase of the study. The planned interim analysis was performed after 31 patients completed the letrozole 5 mg/day intervention. At this time, the futility boundary was crossed, and the study was terminated. While the interim analysis was ongoing, three additional patients completed the interventional treatment period. These patients were not included in the interim analysis, but they contributed to adverse events and quality of life data.

**Fig. 2:**
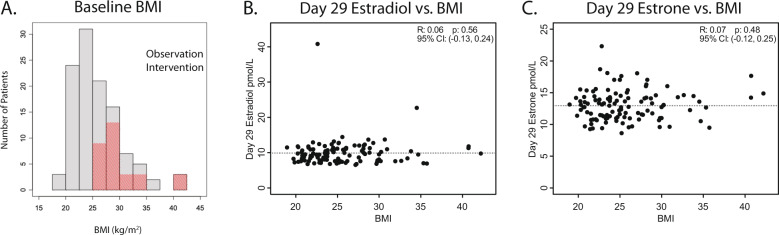
Relationship between individual participant estradiol or estrone levels and BMI. **a** Baseline BMI (kg/m^2^) distribution of all patients (gray, *n* = 112) and those with BMI > 25 kg/m^2^ who continued to the second 28-day interventional period (red, *n* = 31) **b** Association of day 29 estradiol with baseline BMI. **c** Association of day 29 estrone with baseline BMI. Dashed line represents mean value.

The median age at baseline (day 1) of the 112 patients who completed the first 28 days (all participants) was 61 years (Table 1, range 45–79) and median BMI was 24.7 kg/m^2^ (Fig. 2a, Table 1; range 19–42.2 kg/m^2^). The median age of menopause was 50 years (range 31–58 years). The median age of the 31 participants who completed interventional treatment (BMI > 25 kg/m^2^) at the interim analysis was 61 years (range 48–76 years) and median BMI was 28.3 kg/m^2^ (Fig. 2a, Table 1; range, 25.2–42.2 kg/m^2^). 119 out of 112-women enrolled in the study were taking vitamin D supplements (Table 1; range 200 IU–5600 IU/day). The mean time on letrozole prior to study entry was 18.1 months (SD = 17.6, range 3–93; Table 1). Demographic, lifestyle, tumor and treatment characteristics are provided in supplementary Tables S1–3.

**Table 1:** Baseline patient characteristics of patients.

Characteristic	Observation (Day 1–28; *n* = 112)	Intervention (Day 29–58; *n* = 31)
*Age (years)*		
Mean (SD)	61.7 (7.6)	61 (6.6)
Median (Range)	61 (45–79)	61 (48–76)
*Baseline BMI (kg/m*^*2*^*)*		
Mean (SD)	25.7 (4.6)	29.7 (4.5)
Median (Range)	24.7 (19–42.2)	28.3 (25.2–42.2)
*Age at menopause (years)*		
Mean (SD)	49.2 (4.7)	48.4 (5.2)
Median (Range)	50 (31–58)	50 (36–56)
*Vitamin D Supplementation*		
Number of participants (Percent of total)	109 (97%)	31 (100%)
*Letrozole use prior to study (months)*		
Mean (SD)	18.1 (17.6)	
Median (Range)	12.8 (3–93)	

Mean and median characteristics for 112 women participating (observational arm, irrespective of BMI) and 31 women with BMI > 25 kg/m^2^ (interventional arm).

### Relationship of estradiol and estrone with BMI

Day 1 estradiol and estrone levels represent typical usage of prescribed letrozole (generic or branded), as patients were taking letrozole for a minimum of 3 months prior to study enrollment. Day 29 represents four weeks of documented adherence to standardized letrozole on study. Mean estradiol levels were similar at 10.3 pmol/L (SD = 4.24, range 5.0–42.7) on day 1 versus 9.9 pmol/L (SD = 3.73, range 6.4–40.8) on day 29 (Supplementary Fig. 1A; *p* = 0.39). For estrone, mean day 1 levels were 14.6 pmol/L (SD = 15.4, range 9.0–175.3) versus 13.0 pmol/L (SD = 2.4, range 8.6–22.7) on day 29 (Supplementary Fig. 1B; *p* = 0.26). When day 29 estradiol and estrone were plotted against BMI, there was no discernable relationship (Fig. 2b, c). The Pearson correlation coefficient between log-transformed BMI and day 29 estradiol was 0.06 (95% CI = −0.13 to 0.24, *p* = 0.56) and between BMI and day 29 estrone was 0.07 (95% CI = −0.12 to 0.25, *p* = 0.48).

### Relationship of estradiol and estrone with 25-OH vitamin D and markers of inflammation or obesity

**Fig. 3:**
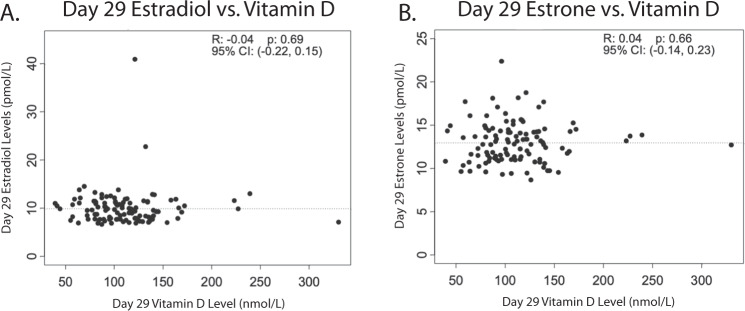
Relationship between individual participant estradiol or estrone levels and 25-OH vitamin D. **a** Association of day 29 estradiol with serum 25-OH vitamin D levels. **b** Association of day 29 estrone with 25-OH vitamin D levels. Dashed line represents mean value.

The mean level of serum 25-OH vitamin D on day 29 was 112 nmol/L (SD = 40.9). When classified by the Institute of Medicine guidelines for 25-OH vitamin D levels^16^, no patients were deficient in 25-OH vitamin D (<30 nmol/L), 3 (2.7%) were insufficient (30–50 nmol/L), 76 (68.5%) had adequate levels (>50 nmol/L), and 32 (28.8%) had levels potentially associated with adverse events (>125 nmol/L)^16^. When day 29 estradiol and estrone were plotted against 25-OH vitamin D levels, there was no discernable trend (Fig. 3a, b). The Pearson correlation coefficient between log-transformed 25-OH vitamin D and day 29 estradiol was −0.04 (95% CI = −0.22 to 0.15, *p* = 0.69) and day 29 estrone was 0.04 (95% CI −0.14 to 0.23, *p* = 0.66).

Several markers of obesity and inflammation, including CRP (marker of inflammation), insulin (marker of insulin resistance), and leptin (marker of adiposity) were measured to evaluate their potential associations with estradiol or estrone levels. Day 29 correlations were not significant, ranging from −0.15 to 0.12 (Supplementary Fig. 1C; *p* = 0.11–0.82).

### Effect of increased dose letrozole on estradiol and estrone levels

Adherence to the 5 mg/day regimen was excellent (participants received 99% of planned doses). Two patients were excluded from further analysis; one had a technical assay failure and one had estradiol and estrone levels inconsistent with menopause at baseline.

**Fig. 4:**
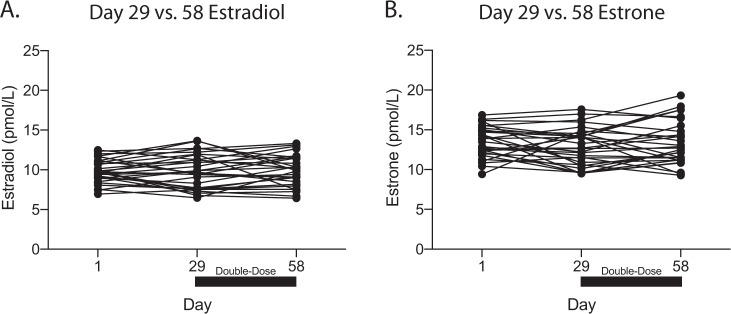
Estradiol and estrone levels after treatment with letrozole 5 mg/day. **a** Individual participant estradiol levels at day 1 (10.32 pmol/L, SD = 4.24), 29 (9.80 pmol/L, SD = 2.16), and 58 (9.91 pmol/L, SD = 1.96). No difference was observed with the letrozole 5 mg/day dose (*p* = 0.74) **b** Individual participant estrone levels at day 1 (14.63 pmol/L, SD = 15.45), 29 (12.93 pmol/L, SD = 2.35), and 58 (13.29 pmol/L, SD = 2.72). No difference was observed with the letrozole 5 mg/day dose (*p* = 0.58).

After the double-dose of letrozole, mean estradiol concentrations were not significantly changed (Fig. 4a; 9.80–9.91 pmol/L, mean change of 0.1 pmol/L, SD = 1.77, 95% CI = −0.56 to 0.78, *p* = 0.74). Similarly, estrone was unchanged (Fig. 4b; 12.9–13.3 pmol/L, mean change of 0.4 pmol/L, SD = 2.2, 95% CI = −0.45 to 1.18, *p* = 0.36). Based on these findings the pre-specified futility boundary for estradiol was crossed and the study was terminated.

Adverse events were infrequent amongst the 34 patients who completed the second 28-day period (Supplementary Table 5). Apart from arthralgia, which was reported more frequently by participants during the double dose intervention, the rates of adverse events were not different between the 2.5 and 5 mg/day doses (Supplementary Table 4). The standardized effect sizes for changes in quality of life symptom scores (BPI and FACT-ES) were all below 0.2, indicating that any changes in this interventional period were small (Supplementary Table 5)^17^.

## Discussion

Optimizing the delivery of proven, standard therapies may improve outcomes for women with early stage breast cancer. Previous data has suggested that women with obesity^18^ or with elevated 25-OH vitamin D levels^15^ may derive less benefit from AIs, identifying an opportunity to tailor therapy based on easily measured host factors.

In all patients, there was no significant correlation between BMI and day 29 serum estradiol or estrone levels under ideal use. In addition to BMI, the lack of correlation between markers of inflammation (CRP), insulin resistance (insulin), and increased adipose tissue (leptin), provided further evidence that poorer outcomes associated with elevated BMI are not due to increased circulating levels of estrogens as a result of peripheral aromatization in fatty tissues and inadequate suppression of aromatase by standard dosing of AI therapy^5^. These findings indicate that even in patients who are overweight/obese or with elevated surrogate serum markers of the metabolic syndrome, letrozole at the standard dose 2.5 mg/day achieves systemic estradiol and estrone suppression, reflecting adequate aromatase inhibition at extremes of BMI. Since all participants were taking letrozole at the time of study entry, it is unknown whether the degree of estrogen suppression from baseline (not receiving AI) is associated with markers of adiposity (BMI, CRP, insulin, or leptin) or 25-OH vitamin D level.

While no association between day 29 estrogens and BMI was observed, it remained possible that higher doses of AI could further suppress estrogen levels in women with elevated BMI (>25 kg/m^2^). However, we found that an increased dose of letrozole (5 mg/day) was not associated with further suppression of serum estrogen levels after 4 weeks with excellent adherence. While the BMI of participants spanned a broad range, few women in the extremely high BMI range (BMI > 35 kg/m^2^) were included, and thus the results of this study cannot necessarily be extrapolated to women with severe obesity.

This study also prospectively evaluated an association between serum 25-OH vitamin D, estradiol, and estrone levels. This study was a follow-up of data from a retrospective cohort study that suggested the biologically plausible notion that serum 25-OH vitamin D could impact the pharmacology of aromatase inhibitors, leading to elevated residual estradiol or estrone^15^. Here we observed no significant correlation between serum 25-OH vitamin D, estradiol or estrone levels in a population with high rates of adequate (>50 nmol/L) and supratherapeutic (>125 nmol/L) levels of vitamin D.

Our study was designed to provide a definitive assessment of estradiol and estrone in women taking adjuvant letrozole under optimal conditions and has several limitations. Participants were enrolled in a small number of centers and were predominantly Caucasian (Supplementary Table 1). In addition, patients tolerating letrozole for at least 3 months prior to enrollment were intentionally recruited in order to examine these relationships under optimal adherence. Given this, it cannot be determined whether lower levels of adherence would achieve similarly complete estradiol or estrone lowering for women irrespective of BMI, CRP, insulin, leptin, or 25-OH vitamin D levels, or whether higher prescribed doses of letrozole could result in improved estrogen lowering in women with BMI > 25 kg/m^2^ under conditions of suboptimal adherence. This limitation is particularly true for women with severe obesity, as few women with BMI > 40 kg/m^2^ were included in our population. Furthermore, the specific study of letrozole limits the generalizability of our results to other AIs. While a previous smaller study showed a statistically significant relationship between BMI and suppressed estrogen levels in women taking letrozole, letrozole is known to reduce estrogens to lower levels than anastrozole in women across a range of BMI^5^. Our data provides reassurance to clinicians that letrozole at its current dosing strategy, with optimal adherence, is sufficient to provide maximum estrogen suppression in women with BMI in the overweight and non-severe obese range.

In conclusion, we found no association between serum 25-OH vitamin D, BMI or related markers (insulin, CRP, and leptin) and residual levels of circulating estrogens in a population of women taking standard dose letrozole therapy. Furthermore, an increased dose of letrozole was not associated with further estrogen lowering in women with BMI > 25 kg/m^2^. These results support the appropriateness of the standard 2.5 mg/day letrozole dose in postmenopausal women who are overweight or have moderate levels of obesity.

## Methods

### Study design

A prospective study was performed in postmenopausal women with hormone receptor positive early breast cancer. Participants were receiving adjuvant letrozole therapy for a minimum of 3 months at study enrollment. All the participants provided written, informed consent before trial entry. All women were included in day 1–28 of the study, which consisted of study visits at day 1 and day 29. Participants were provided with a 28-day standard supply of branded letrozole (Femara, 2.5 mg/day) and instructed to take the drug at 10 a.m., keeping a medication journal. On study entry and day 29, a fasting blood sample was taken prior to letrozole consumption. Consenting participants with a BMI > 25 kg/m^2^ then received a double dose of letrozole (5 mg/day) for an additional 28 days, followed by a day 58 fasting blood sample. The study was approved by the Ontario Research Ethics Board (OCREB), as well as each participating center. Use of the letrozole 5 mg dose was authorized by a Health Canada Clinical Trial Application. The study was registered on August 21st, 2012 with ClincialTrials.gov [NCT01669343].

### Ethics approval and consent to participate

This trial received ethics approval from Research Ethics Board of the University Health Network, Mount Sinai Hospital, Women’s College Hospital, St. Michael’s Hospital, Sunnybrook Hospital, and the Ontario Cancer Ethics Review Board (OCREB). All participants enrolled in the study provided informed consent to be enrolled into the study and to have the results of the study disseminated through academic publication. Consent was obtained in accordance with institutional ethics board guidelines.

### Patient selection

All participants had a confirmed histological diagnosis of ER and/or PR positive breast cancer (Stage I-III) and had completed local therapy prior to study enrollment. Patients had to be menopausal at study entry, defined as: (i) no menstrual period in the past 12 months (ii) previous bilateral oophorectomy with or without hysterectomy at any age or (iii) previous hysterectomy without oophorectomy and age greater than 55. Patients with known persistent local or metastatic cancer, liver dysfunction (AST or ALT 1.5× institutional limit of normal), or significant renal dysfunction (estimated Cr clearance <40 mL/min) were excluded.

### Safety monitoring

All participants were actively taking and tolerating letrozole 2.5 mg/day for at least 3 months at the time of study entry. Adverse events were collected at regular study visits and at follow-up telephone calls. Potential AI-associated toxicities were evaluated using validated self-administered quality of life questionnaires including: functional assessment of cancer therapy: endocrine symptoms (FACT-ES)^19^ and brief pain inventory-short form interference and pain (BPI-SF pain, BPI-SF interference, musculoskeletal symptoms)^20^.

### Measurement, blood collection, and assays

Participants provided demographic information at study enrollment. Oncologic history and tumor pathology were extracted from medical records. Vitamin D supplement use, medications and hormone replacement therapy were documented at all three study visits. At days 29 and 58, study drug adherence was recorded based on medication journal and pill counts.

Blood samples were processed and stored centrally until batched analyses were performed. High sensitivity estrogen assays were performed via solvent extraction-column chromatography-RIA^21^. This assay has an E2 lower limit of detection of detection of 1.8 pg/mL (6.6 pmol/L), providing necessary sensitivity for the measurement of estrogen levels in the AI-treated population. Plasma 25-OH vitamin D (Roche), insulin (Roche), insulin (Roche), and leptin (Quantikine) were measured by a standard chemiluminescent immunoassay.

### Statistical analysis

A priori power calculations indicated 106 patients were needed to have ≥84% power to detect a 20% increase in estradiol in all patients enrolled as (i) BMI increased from 25 to 30 kg/m^2^ or (ii) 25-OH vitamin D increased from 50 to 72 nmol/L. More patients were enrolled to allow for drop-out. The associations of day 29 estradiol (main outcome) and estrone with BMI and 25-OH vitamin D were examined via graphs and Pearson correlation coefficients as summary measures. Associations with CRP, insulin and leptin were assessed in a similar fashion (secondary analyses). The log-scale was pre-specified because of skew distributions observed in previous studies. Paired *t*-tests were used to test for equality of means between different days.

We planned to enroll 62 patients with BMI > 25 kg/m^2^ who would continue to the interventional (letrozole 5 mg/day) portion of the study. An interim analysis for both efficacy and futility was performed when 31 patients completed the double dose. We used a paired *t*-test to compare days 29–58 estradiol levels and the t-statistic *t* to define the efficacy and futility boundaries (futility boundary |*t*| = 0.5 provided a low probability (5%) for wrongly stopping the test for futility if there really is a change in estradiol (set at ≥18%), and efficacy boundary |*t*| = 2.96 provided a low probability (0.5%) of wrongly stopping the test for efficacy if there really was no change in estradiol.

Adverse events were graded as per NCI CTCAE v4.01 and tabulated. Changes in the quality of life indices were expressed as standardized effect sizes, calculated as the difference in mean scores divided by the standard deviation at the start day.

### Reporting summary

Further information on research design is available in the Nature Research Reporting Summary linked to this article.

## Supplementary information

Reporting Summary

Supplementary Information

## Data Availability

The data generated and analysed during this study are described in the following data record: 10.6084/m9.figshare.12192042^22^. Data files are available upon reasonable request to the corresponding author.
