# Supplementary material for: Association between BMI, vitamin D, and estrogen levels in postmenopausal women using adjuvant letrozole: a prospective study
Source: NPJ Breast Cancer. 2020 Jun 12;6:22. doi: 10.1038/s41523-020-0166-y (PMC7293309; doi:10.1038/s41523-020-0166-y)
Supplement: Supplementary file 2 — Supplementary Information [file 41523_2020_166_MOESM2_ESM.pdf]

Association between BMI, vitamin D, and estrogen levels in postmenopausal women using adjuvant letrozole: A prospective study

Mitchell J. Elliott [1,2], Marguerite Ennis [3], Kathleen I. Pritchard [2,4], Carol Townsley [5], Dave Warr [1,2], Christine Elser [1,2,6], Eitan Amir [2,6], Philippe L. Bedard [1,2], Lakshmi Rao [6], Vuk Stambolic [1,2], Srikala Sridhar [1,2], Pamela J. Goodwin [1,6,7], David W. Cescon [1,2,*]

[1]Princess Margaret Cancer Centre, Toronto, Canada

[2]University of Toronto, Toronto, Canada

[3]Applied Statistician, Markham, Canada

[4]Sunnybrook Odette Cancer Centre, Toronto, Canada

[5]Women's College Hospital, Toronto, Canada

[6]Marvelle Koffler Breast Centre, Mount Sinai Hospital, Toronto, Canada

[7]Lunenfeld Tanenbaum Research Institute, Mt. Sinai Hospital, Toronto, Canada

[*]dave.cescon@uhn.ca

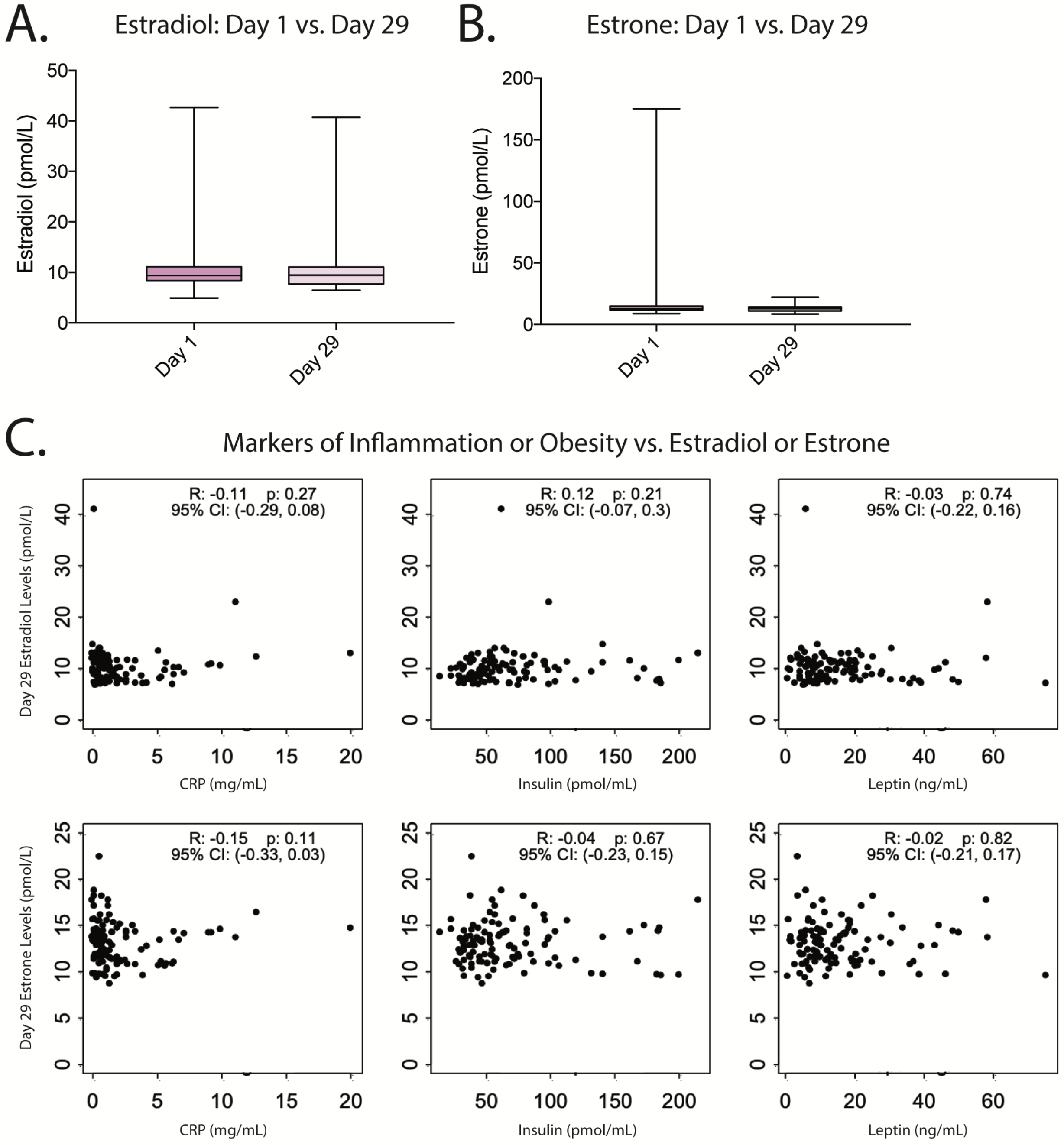

Supplementary Figure 1. (A) Box and whisker plot representing median, first and third quartiles and minimum and maximum values of estradiol or (B) estrone in 112 women at day 1 (typical use of letrozole) and day 29 (2.5 mg/day of monitored letrozole use) (C) CRP, insulin, and leptin are markers of inflammation, insulin resistance, and adiposity, respectively. Correlation between these markers and estradiol or estrone.

Characteristic	Observation (Day 1-28; n=112)	Intervention (Day 29-58; n=31)
Untanned skin colour		
Very Fair/Fair	76 (67.9%)	18 (58.1%)
Medium/Olive	30 (26.8%)	9 (29%)
Brown/Very Dark	6 (5.4%)	4 (12.9%)
Baseline glucose (mmol/L)		
Mean (SD)	5.6 (1.2)	5.9 (1.9)
Median (Range)	5.3 (4.5 - 14.5)	5.4 (4.6 - 14.5)
Previous or ongoing smoking		
Yes	42 (37.5%)	12 (38.7%)
No	70 (62.5%)	19 (61.3%)
Previous or ongoing alcohol consumption		
Yes	56 (50%)	14 (45.2%)
No	56 (50%)	17 (54.8%)

Supplementary Table 1. Additional baseline characteristics of patients. Data obtained from standardized entrance questionnaire and baseline bloodwork at study enrollment.

Characteristic	Observation (Day 1-28; n=112)	Intervention (Day 29-58; n=31)
T-stage		
T1	46 (41.1%)	11 (35.5%)
T2	46 (41.1%)	16 (51.6%)
T3	15 (13.4%)	3 (9.7%)
T4	3 (2.7%)	1 (3.2%)
TX	2 (1.8%)	0 (0%)
N-stage		
N0	59 (52.7%)	14 (45.2%)
N1	34 (30.4%)	10 (32.3%)
N2	11 (9.8%)	4 (12.9%)
N3	8 (7.1%)	3 (9.7%)
Histological diagnosis		
Ductal	83 (74.1%)	27 (87.1%)
Lobular	13 (11.6%)	2 (6.5%)
Mixed	13 (11.6%)	2 (6.5%)
Other	3 (2.7%)	0 (0%)
Tumour Grade		
Grade I	10 (8.9%)	1 (3.2%)
Grade II	71 (63.4%)	20 (64.5%)
Grade III	29 (25.9%)	10 (32.3%)
Unknown	2 (1.8%)	0 (0%)
Lymphovascular invasion		
Positive	56 (50%)	19 (61.3%)
Negative	53 (47.3%)	12 (38.7%)
Unknown	3 (2.7%)	0 (0%)
Estrogen Receptor		
Negative	0 (0%)	0 (0%)
Positive	112 (100%)	31 (100%)
Progesterone Receptor		
Negative	20 (17.9%)	3 (9.7%)
Positive	92 (82.1%)	28 (90.3%)
HER2 Receptor		
Negative	100 (89.3%)	27 (87.1%)
Positive	12 (10.7%)	4 (12.9%)

Supplementary Table 2. Patient tumor characteristics. Data obtained from clinical and pathological staging as reported by central confirmation of pathology specimen acquired via the patient electronic medical record.

Characteristic	Observation (Day 1-28; n=112)	Intervention (Day 29-58; n=31)
Surgery		
Lumpectomy	65 (58%)	21 (67.7%)
Mastectomy	47 (42%)	10 (32.3%)
Radiation		
No	15 (13.4%)	2 (6.5%)
Yes	97 (86.6%)	29 (93.5%)
Chemotherapy		
None	37 (33%)	9 (29%)
FEC-Docetaxel	46 (41.1%)	16 (51.6%)
TC	17 (15.2%)	4 (12.9%)
AC-Taxol	8 (7.1%)	2 (6.5%)
Other	4 (3.6%)	0 (0%)
Herceptin		
No	100 (89.3%)	27 (87.1%)
Yes	12 (10.7%)	4 (12.9%)
Tamoxifen		
No	44 (39.3%)	15 (48.4%)
Yes	68 (60.7%)	16 (51.6%)

Supplementary Table 3. Patient treatment characteristics. Data was obtained from patient treatment history extracted from patient electronic medical record.

Adverse Events				
	Observation (Day 1-28; n=121)		Intervention (Day 29-58; n=31)	
	Grade 1 or 2	Grade 3	Grade 1 or 2	Grade 3
Abdominal Pain	1	-	-	-
Amnesia	-	-	1	-
Anemia	-	-	1	-
Arthralgia	1	-	6	1
Bloating	-	-	1	-
Breast Infection	-	-	-	1
Cardiac Disorder	-	-	1	-
Conjunctivitis	1	-	-	-
Constipation	-	1	1	-
Cough	4	-	-	-
Diarrhea	1	-	2	-
Dyspnea	1	-		-
Epistaxis		-	1	-
Fatigue	1	-	2	-
Flu-like Symptoms	2	-	-	-
Headache	1	-	-	1
Infection (other)	-	-	1	-
Insomnia	-	-	1	-
Myalgia	-	-	2	-
Nausea	1	-	1	-
Premature Menopause	-	-	2	-
Renal Colic	-	1	-	-
Shingles	-	-	1	-
Sinusitis	1	-	-	-
Stroke	1	-	-	-
Tooth Infection	1	-	-	-
Upper Respiratory Infection	1	-	1	-
Urinary Tract Infection	1	-	1	-
Uterine Hemorrhage	1	-	1	-

Supplementary Table 4. Adverse Events Type. List of adverse events observed during the trial. Events are separated by event type and grade.

	Observational Cohort Completers (n=112)				Interventional Cohort Completers (n=34)			
	Day 1 SD	Day 1 Mean	Day 29 Mean	stES	Day 29 SD	Day 29 Mean	Day 58 Mean	stES
BPI-SF Pain	1.66	1.5	1.64	0.08	2.24	2.13	2.03	-0.05
BPI-SF Interference	1.48	0.99	1.26	0.18	2.54	1.92	1.63	-0.11
FACT-ES	9.55	62.41	62.86	0.05	12.91	59.52	59.39	-0.01

Supplementary Table 5. Change in quality of life indices during day 1 to 28 and during day 29 to 58. The standardized effect size (stES) for change is the difference in mean scores divided by the standard deviation (SD) at the start day. The indices are the pain and interference scores of the BPI-SF (Brief Pain Inventory, Short Form) and the FACT-ES (Functional Assessment of Cancer Therapy – Endocrine Score) score.
